# Social Determinants of Health and Cardiologist Involvement in the Care of Adults Hospitalized for Heart Failure

**DOI:** 10.1001/jamanetworkopen.2023.44070

**Published:** 2023-11-20

**Authors:** David T. Zhang, Chukwuma Onyebeke, Musarrat Nahid, Lauren Balkan, Mahad Musse, Laura C. Pinheiro, Madeline R. Sterling, Raegan W. Durant, Todd M. Brown, Emily B. Levitan, Monika M. Safford, Parag Goyal

**Affiliations:** 1 Department of Medicine, Weill Cornell Medicine, New York, New York; 2 Department of Health Policy and Management, Weill Cornell Medicine, New York, New York; 3 Department of Medicine, University of Alabama at Birmingham, Birmingham; 4 Department of Epidemiology, University of Alabama at Birmingham, Birmingham; 5 Program for the Care and Study of the Aging Heart, Weill Cornell Medicine, New York, New York

## Abstract

**Importance:**

Involvement of a cardiologist in the care of adults during a hospitalization for heart failure (HF) is associated with reduced rates of in-hospital mortality and hospital readmission. However, not all patients see a cardiologist when they are hospitalized for HF.

**Objective:**

To determine whether social determinants of health (SDOH) are associated with cardiologist involvement in the management of adults hospitalized for HF.

**Design, Setting, and Participants:**

This retrospective cohort study used data from the Reasons for Geographic and Racial Difference in Stroke (REGARDS) cohort. Participants included adults who experienced an adjudicated hospitalization for HF between 2009 and 2017 in all 48 contiguous states in the US. Data analysis was performed from November 2022 to January 2023.

**Exposures:**

A total of 9 candidate SDOH, aligned with the Healthy People 2030 conceptual model, were examined: Black race, social isolation, social network and/or caregiver availability, educational attainment less than high school, annual household income less than $35 000, living in rural area, living in a zip code with high poverty, living in a Health Professional Shortage Area, and living in a state with poor public health infrastructure.

**Main Outcomes and Measures:**

The primary outcome was cardiologist involvement, defined as involvement of a cardiologist as the primary responsible clinician or as a consultant. Bivariate associations between each SDOH and cardiologist involvement were examined using Poisson regression with robust SEs.

**Results:**

The study included 1000 participants (median [IQR] age, 77.8 [71.5-84.0] years; 479 women [47.9%]; 414 Black individuals [41.4%]; and 492 of 876 with low income [56.2%]) hospitalized at 549 unique US hospitals. Low annual household income (<$35 000) was the only SDOH with a statistically significant association with cardiologist involvement (relative risk, 0.88; 95% CI, 0.82-0.95). In a multivariable analysis adjusting for age, race, sex, HF characteristics, comorbidities, and hospital characteristics, low income remained inversely associated with cardiologist involvement (relative risk, 0.89; 95% CI, 0.82-0.97).

**Conclusions and Relevance:**

This cohort study found that adults with low household income were 11% less likely than adults with higher incomes to have a cardiologist involved in their care during a hospitalization for HF. These findings suggest that socioeconomic status may bias the care provided to patients hospitalized for HF.

## Key Points

**Question:**

Are social determinants of health associated with cardiologist involvement in the management of adults hospitalized for heart failure?

**Findings:**

This cohort study of 1000 participants hospitalized for heart failure at 549 unique US hospitals found that low income was inversely associated with cardiologist involvement after adjusting for patient-based and hospital-based factors.

**Meaning:**

These findings identify socioeconomic status as an important social determinant of health that may bias the care provided to patients hospitalized for heart failure.

## Teaser

This cohort study examines whether social determinants of health are associated with cardiologist involvement in the management of adults hospitalized with heart failure (HF).

## Introduction

Heart failure (HF) is the one of the most common reasons for hospitalization among adults, with high rates of readmission, health care costs, and mortality.^1,2,3,4,5^ The involvement of a cardiologist in the care of adults hospitalized for HF is associated with reduced rates of readmission and mortality.^6,7,8,9,10,11,12,13,14,15,16^ Yet, cardiologist involvement is not universal when adults are hospitalized for HF. Some of this might relate to cardiologist availability, which is especially problematic in rural settings with shortages of cardiologists.^2,7^

However, another potential contributor may be implicit bias. Indeed, in 2 studies^17,18^ of adults hospitalized with HF, Black race was associated with decreased odds of cardiologist involvement in the intensive care unit (ICU) and on the floor. In another single-center study,^19^ both Black and Latinx adults hospitalized for HF were less likely than White adults to be admitted to a cardiology service and subsequently experienced worse outcomes. Differential utilization of cardiologists in the management of HF is not unique to race.^20,21,22^ In a multicenter study, Auerbach et al^17^ found that adults admitted with severe HF who had an annual income less than $11 000 were less likely to receive care from a cardiologist. These observations raise concern that social determinants of health (SDOH), an increasingly recognized contributor to health care delivery,^23,24^ lead to suboptimal care in the management of adults hospitalized for HF.

Notably, prior studies on this topic have not examined multiple SDOH concurrently and have lacked geographic diversity. With the current study, we sought to investigate the association between 9 different candidate SDOH and the involvement of cardiologists in the care of adults hospitalized for HF, using the Reasons for Geographic and Racial Differences in Stroke (REGARDS) cohort, which represents a geographically diverse cohort, includes HF hospitalizations from more than 500 unique hospitals, and offers access to a broad range of SDOH.^25,26,27,28,29,30,31,32^

## Methods

The protocol for this cohort study was reviewed and approved by Weill Cornell Medicine’s and the University of Alabama at Birmingham’s institutional review boards. All participants provided written informed consent. This study followed the Strengthening the Reporting of Observational Studies in Epidemiology (STROBE) reporting guidelines for cohort studies.

### Study Population

The study population included adults from REGARDS with an adjudicated HF hospitalization (as principal reason for hospitalization) in 2009 to 2017. In brief, the REGARDS cohort originally included 30 239 community-dwelling Black and White men and women aged 45 years or older recruited via mailing followed by telephone contact in 2003 to 2007 from all 48 contiguous states in the US and the District of Columbia to investigate racial and geographic disparities in stroke mortality.^33^ Black adults and residents of the so-called Stroke Belt (ie, Alabama, Arkansas, Georgia, Louisiana, Mississippi, North Carolina, South Carolina, and Tennessee), an area in the southeastern US with high stroke mortality, were oversampled by design; this includes a 153-county region in Georgia, North Carolina, and South Carolina known as the Stroke Buckle, which has even higher rates of stroke compared with the rest of the Stroke Belt. Participants were recruited using commercially available lists. They underwent an initial 45-minute computer-assisted telephone interview about medical history, health behaviors, and risk factors followed by an in-home visit for baseline vital signs, electrocardiograms, medication reconciliation, bloodwork, and urine samples. At 6-month intervals, participants were contacted by telephone and asked about their health status and study outcomes such as hospitalizations. When participants reported a hospitalization for a possible cardiovascular cause, their medical records were retrieved and underwent review and adjudication. HF adjudication was conducted by 2 expert clinicians (not coauthors of the present article) on the basis of medical record–level data on symptoms, physical examinations, laboratory values, imaging, and medical treatments. In cases of a disagreement, a committee discussed to reach a consensus on a final decision.

For this study, we excluded participants hospitalized at institutions that lack cardiology services according to linked American Hospital Association data.^34^ Of note, there were hospitals included in the American Hospital Association data set that lacked information on the availability of cardiology services. To mitigate risk of bias related to excluded patients hospitalized at these hospitals, we imputed values (for 176 participants) for the availability of cardiology services using a random forest method that incorporated most other American Hospital Association data (including, but not limited to, location, bed size, teaching status, geriatrics service, palliative care service, and licensed pharmacists). To do this, we used the R package missForest.^35,36^ We examined each participant’s first adjudicated hospitalization during the study period.

### Exposure: SDOH

The candidate exposures were 9 SDOH available in REGARDS,^25,26,27,28,29,30,31,32^ following the Healthy People 2030 conceptual framework.^37^ This framework includes 5 domains: economic stability, education access and quality, social and community context, health care access and quality, and neighborhood and built environment.^37^ eFigure 1 in Supplement 1 outlines the SDOH from REGARDS mapped onto the Healthy People 2030 conceptual framework.

Similar to prior work,^25,26,27,28,29,30,31,32^ we examined (1) Black race, (2) social isolation (defined as having 0-1 visits from a family or friend in the past month), (3) social network and/or caregiver availability (defined as whether the participant reported having someone to care for them if ill), (4) low educational attainment (less than high school education), (5) low annual household income (<$35 000),^38^ (6) living in rural areas (defined as living in an isolated or small rural area on the basis of Rural Urban Commuting Area Codes), (7) living in a zip code with high poverty (>25% of residence below the federal poverty level), (8) living in a Health Professional Shortage Area, and (9) living in a state with poor public health infrastructure (assessed using data from the America’s Health Ranking,^39^ which ranked states according to their contribution to lifestyle, access to care, and disability; states where REGARDS participants lived that fell into the bottom 20th percentile for their ranking for ≥8 of the 10 years spanning 2000 to 2010, concordant with REGARDS recruitment between 2003 to 2007, were considered to have poor public health infrastructure).

### Outcome

The primary outcome was cardiologist involvement, which was defined as involvement of a cardiologist as the primary responsible clinician or as a consultant. This variable was collected through a rigorous medical record review and abstraction process previously described.^40^ Medical record review and abstraction involved review of admission notes, progress notes, consultation notes, discharge summaries, medication reconciliations, laboratory values, and imaging studies to collect relevant data related to the HF hospitalization including cardiologist involvement.

### Covariates

Covariates were selected on the basis of the Andersen Behavioral Model, which outlines predisposing, enabling, and need factors that contribute to health care utilization.^41^ Because the primary exposures (SDOH) are considered enabling factors, covariates included predisposing factors and need factors. Predisposing factors included age (at time of admission) and baseline self-reported race, sex, and an indicator for Stroke Belt residence. Need factors included the following 3 subcategories: HF characteristics, comorbid conditions, and hospital characteristics. HF characteristics and comorbid conditions were collected according to medical record abstraction, and hospital characteristics were collected according to American Hospital Association survey data, which have been linked to the REGARDS cohort. HF characteristics included New York Heart Association Class (class I-IV), HF subtype (HF with preserved ejection fraction [EF] if EF ≥50% or qualitative description of normal systolic function),^42^ Get With The Guidelines–Heart Failure (GWTG-HF) Risk Score on admission (a score that estimates in-hospital all-cause mortality with a range of 0 to ≥79),^43^ length of stay, ICU stay, and in-hospital cardiac arrest. Comorbid conditions included coronary artery disease, history of arrythmia (atrial and/or ventricular), diabetes, history of stroke, and the number of noncardiac comorbid conditions (>40) spanning multiple organ systems. Hospital characteristics included each hospital’s total number of beds, presence or absence of cardiac ICU, and academic teaching status.

### Statistical Analysis

Data analysis was performed from November 2022 to January 2023. We calculated the frequency and distribution of each SDOH and examined collinearity using φ coefficients (eTable 1 in Supplement 1). We then examined associations between each SDOH and cardiologist involvement using relative risks (RRs). Similar to prior REGARDS studies,^25,26,27,28,29,30,31,32^ candidate SDOH with statistically significant associations (*P* < .10) were retained for further multivariable analysis. All SDOH were collected at the time of initial REGARDS baseline surveys.

To describe patient demographic and clinical characteristics and hospital characteristics, descriptive statistics such as frequencies, percentages, medians, and IQRs were calculated. χ^2^ tests and Mann-Whitney *U* tests were conducted for categorical and continuous variables, respectively, to identify significant differences (*P* < .05) across categories of the retained SDOH.

We then examined the association between retained SDOH and cardiologist involvement by conducting a modified Poisson regression with robust SE, adjusting for the previously described covariates. We chose a modified Poisson regression to estimate RR given the high prevalence of cardiologist involvement.^44^ To examine for a potential interaction by age 65 years or older, we added cross-product terms to the models and performed a Wald test.

We used 2-sided hypothesis testing with *P* < .05 for all analyses performed. Multiple imputation by chained equations was used to impute missing SDOH and covariates to minimize bias under the assumption that data were missing at random. Analyses were performed in Stata statistical software version 17 (StataCorp) and R statistical software version 4.2.3 (R Project for Statistical Computing).

## Results

We examined 1000 participants hospitalized at 549 unique US hospitals, of whom 751 (75.1%) received cardiology services while hospitalized. eFigure 2 in Supplement 1 shows the exclusion cascade. The median (IQR) age of the cohort was 77.8 (71.5-84.0) years, 479 (47.9%) were female, and 414 (41.4%) were Black (Table 1).

**Table 1.** Participant Characteristics

Characteristic	Participants, No. (%)
Age, median (IQR), y (n = 1000)	77.8 (71.5-84.0)
Race (n = 1000)	
Black	414 (41.4)
White	586 (58.6)
Sex (n = 1000)	
Female	479 (47.9)
Male	521 (52.1)
Region (n = 1000)	
Stroke Buckle (153-county region in Georgia, North Carolina, and South Carolina)	218 (21.8)
Stroke Belt (Alabama, Arkansas, Georgia, Louisiana, Mississippi, North Carolina, South Carolina, and Tennessee)	363 (36.3)
Rest of the contiguous US	419 (41.9)
Social determinants of health	
Low annual household income (n = 876)	492 (56.2)
Living in rural areas (n = 900)	25 (2.8)
Living in a zip code with high poverty (n = 991)	218 (22.0)
Living in a Health Professional Shortage Area (n = 1000)	431 (43.1)
Poor public health infrastructure (n = 1000)	384 (38.4)
Social isolation (n = 934)	128 (13.7)
Social network (n = 979)	138 (14.1)
Low educational attainment (n = 1000)	175 (17.5)
HF characteristics	
New York Heart Association Class (n = 763)	
I	54 (7.1)
II	237 (31.1)
III	303 (39.7)
IV	169 (22.1)
HF with preserved ejection fraction (n = 758)	336 (44.3)
Get With The Guidelines–HF Risk Score, median (IQR) (n = 981)	2.0 (2.0-2.0)
Length of stay, median (IQR), d (n = 996)	5.0 (3.0-8.0)
ICU stay (n = 995)	217 (21.8)
In-hospital cardiac arrest (n = 996)	626 (67.1)
Comorbid conditions (n = 1000)	
Coronary artery disease	755 (75.5)
Arrhythmia	443 (44.3)
No. of noncardiac comorbidities, median (IQR)	5.0 (3.0-7.0)
Diabetes	503 (50.3)
Stroke	259 (25.9)
Hospital characteristics	
Total No. of hospital beds, median (IQR) (n = 976)	373.0 (233.0-592.0)
Presence of cardiac ICU (n = 933)	626 (67.1)
Academic hospital (n = 945)	571 (60.4)

Abbreviations: HF, heart failure; ICU, intensive care unit.

**Figure 1.**
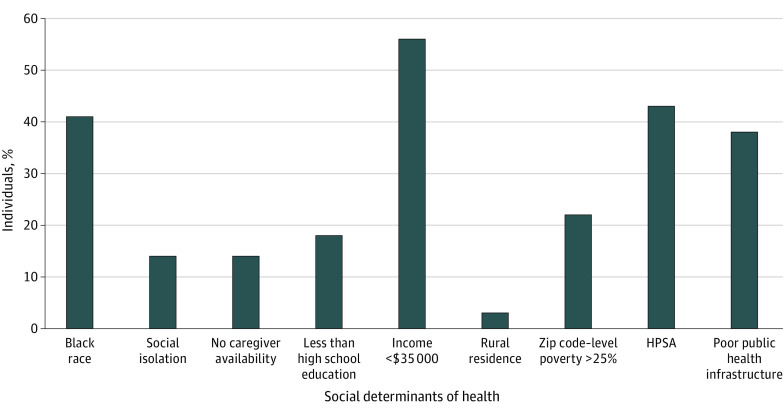
Prevalence of Individual Social Determinants of Health The prevalence of the 9 social determinants of health were calculated among Reasons for Geographic and Racial Difference in Stroke patients. Low annual household income (<$35 000) had the highest prevalence (56.2%) among social determinants of health. HPSA indicates Health Professional Shortage Area.

**Figure 2.**
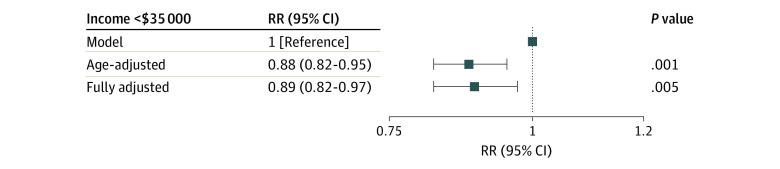
Relative Risk (RR) for Association of Income Less Than $35 000 With Cardiologist Involvement Low income was inversely associated with cardiologist involvement in both age-adjusted analysis (RR, 0.88; 95% CI, 0.82-0.95) and fully adjusted models (RR, 0.89; 95% CI, 0.82-0.97).

The prevalence of each of the 9 candidate SDOH is shown in Figure 1, and baseline variables stratified according to cardiologist involvement are provided in eTable 2 in Supplement 1. The associations between each of the SDOH and cardiologist involvement are shown in Table 2. Low income was the only candidate SDOH with a statistically significant association with cardiologist involvement (age-adjusted RR, 0.88; 95% CI, 0.82-0.95; *P* < .001) (Figure 2).

**Table 2.** Age-Adjusted RR for the Association of Candidate SDOH With Cardiologist Involvement

SDOH	RR (95% CI)
Black race	0.97 (0.90-1.04)
Social isolation	0.96 (0.86-1.07)
Social network	0.91 (0.80-1.03)
Low educational attainment	0.95 (0.86-1.05)
Low annual household income	0.88 (0.82-0.95)^a^
Living in rural areas	0.98 (0.78-1.23)
Living in a zip code with high poverty	0.96 (0.88-1.05)
Living in a Health Professional Shortage Area	1.03 (0.96-1.11)
Poor public health infrastructure	1.03 (0.96-1.11)

Abbreviations: RR, relative risk; SDOH, social determinants of health.

^a^ Retained for further analysis.

Within the cohort, 876 participants had baseline income data. Characteristics of those 876 participants stratified by income are shown in Table 3. The median (IQR) age was 77.5 (71.0-83.7) years, 402 (45.9%) were female, 363 (41.4%) were Black, and 492 (56.2%) had low income (median annual income range, $25 000-$35 000). Participants with low income were more likely to be Black and female and to have diabetes. Those with low income were more likely to have HF with preserved EF, less likely to have a history of arrythmia, and had a lower GWTG-HF Risk Score on admission. Of note, 94.9% of the study cohort (831 participants) had insurance, and the prevalence of having insurance was comparable among those who had and did not have involvement of a cardiologist in their care. The highest rates of missing data were HF subtype (212 participants [24.2%]), New York Heart Association class (207 participants [23.6%]), presence of cardiac ICU (58 participants [6.6%]), and hospital teaching status (50 participants [5.7%]). All other covariates were missing less than 3% of participants.

**Table 3.** Participant Characteristics Stratified by Annual Household Income

Characteristic	Participants, No. (%)		*P* value
	Income ≥$35 000 (n = 384)	Income <$35 000 (n = 492)	
Age, median (IQR), y (n = 876)	77.31 (71.46-83.38)	77.89 (70.45-84.04)	.82
Black race (n = 363)	108 (28.1)	255 (51.8)	<.001
Sex (n = 876)			
Female	115 (29.9)	287 (58.3)	<.001
Male	269 (70.1)	205 (41.7)	
Region (n = 876)			
Stroke Buckle (153-county region in Georgia, North Carolina, and South Carolina)	98 (25.5)	100 (20.3)	.05
Stroke Belt (Alabama, Arkansas, Georgia, Louisiana, Mississippi, North Carolina, South Carolina, and Tennessee)	124 (32.3)	195 (39.6)	
Rest of the contiguous US	162 (42.2)	197 (40.0)	
HF characteristics			
New York Heart Association Class (n = 669)			
I	24 (8.1)	25 (6.7)	.52
II	84 (28.2)	121 (32.6)	
III	120 (40.3)	150 (40.4)	
IV	70 (23.5)	75 (20.2)	
HF with preserved ejection fraction (n= 664)	114 (38.8)	178 (48.1)	.02
Get With The Guidelines–HF Risk Score, median (IQR) (n = 859)	2 (2-2)	2 (1-2)	.03
Length of stay, median (IQR), d (n = 873)	4 (3-8)	5 (3-8)	.96
ICU stay (n = 872)	81 (21.2)	110 (22.4)	.66
In-hospital cardiac arrest (n = 873)	12 (3.1)	23 (4.7)	.25
Comorbid conditions (n = 876)			
Coronary artery disease	295 (76.8)	376 (76.4)	.89
Arrhythmia	194 (50.5)	194 (39.4)	<.001
No. of noncardiac comorbidities, median (IQR)	5 (4-7)	5 (3-7)	.29
Diabetes	166 (43.2)	272 (55.3)	<.001
Stroke	89 (23.2)	130 (26.4)	.27
Hospital characteristics			
Total No. of hospital beds, median (IQR) (n = 854)	384 (237-603)	362 (234-592)	.43
Presence of cardiac ICU (n = 818)	246 (68.9)	305 (66.2)	.41
Academic hospital (n = 826)	222 (62.4)	282 (60.0)	.49

Abbreviations: HF, heart failure; ICU, intensive care unit.

In a fully adjusted model, low income remained inversely associated with cardiologist involvement during hospitalization (adjusted RR, 0.89; 95% CI, 0.82-0.97; *P* < .001) (Figure 2). We did not find a significant interaction with age 65 years or older.

## Discussion

In this cohort study of adults hospitalized for HF, we found that low annual income (<$35 000) was associated with lower likelihood of cardiologist involvement during hospitalization. Prior work has shown the potential influence of SDOH on cardiologist involvement among adults with HF, but studies have been limited regarding the number and breadth of SDOH included. For example, Breathett et al^18^ previously reported that Black race was inversely associated with receiving care from a cardiologist among adults admitted to the ICU with HF. Their study included race but did not include socioeconomic status. Auerbach et al^17^ showed that low income was inversely associated with having a cardiologist as the primary responsible clinician (primary attending physician), but their study only included those with advanced HF with reduced EF and did not adjust for other potential SDOH, such as social isolation, social network, or area of residence. Our study included 9 different SDOH (including race and income) that span multiple domains and examined adults from more than 500 hospitals across the US across the entire spectrum of left ventricular EF and severity. Moreover, our study uniquely examined involvement of cardiologists as either the primary responsible clinician or a consultant; prior work to date has primarily examined factors associated with having a cardiologist as the primary responsible clinician. Accordingly, our study now provides one of the most comprehensive examinations of SDOH and cardiologist involvement to date.

Race is a well-established critical factor associated with bias and disparities in health outcomes, including for cardiac therapeutic options and outcomes.^45^ Interestingly, race did not emerge as a significant factor in our study examining the involvement of a cardiologist in the care of adults hospitalized for HF. Although this should not alter our conceptual understanding of how race affects health outcomes, our findings should serve as a reminder that there are other SDOH that are important and play critical roles in clinician behavior, care processes, and health outcomes. Our findings here further highlight the value of examining a broad range of SDOH to optimally understand the impact that these factors have within health care.^46^ The field would also benefit from a deeper examination of mediating factors such as discrimination, allostatic load, housing instability, and childhood exposures, to name a few.

Our observations remained even after controlling for length of stay, severity of disease, HF subtype, and hospital characteristics. This raises concern that our observation could relate to implicit or even explicit bias. Implicit bias is the subconscious stereotype or attitude that consequentially affects behavior and actions, which can contribute to health care disparities.^47,48^ The concept of SDOH contributing to implicit bias with a subsequent effect on care provision is not new.^16,49,50^ However, this is one of the first studies to suggest that low income can contribute to implicit bias.^50^ Because income status may not be readily apparent to clinicians, further investigation is needed to better understand whether specific patient attributes or behaviors contribute to the suspected implicit bias observed among adults with low income. There is also a possibility of explicit bias, whereby clinician behavior may be directly impacted by awareness of the patient’s income status and related notions about treatment adherence and/or affordability of services for adults with low income. Developing treatment strategies that incorporate social factors is important, but systematically withholding therapies can lead to worsening of disparities. Our observations could also be explained by patients themselves requesting a cardiology consultation; whether patients with specific SDOH such as low income are less likely to request involvement of a specialist is not known and merits additional investigation. An important factor that may mediate these findings is insurance status. Although most participants in this cohort had insurance, we were unable to differentiate patients with different types of insurance. Future work should examine whether insurance type and related concepts such as network participation and cost-sharing are contributors to our findings. It is also not known whether lower rates of cardiologist involvement mediate the well-described associations between low socioeconomic status and hospital readmission or mortality among adults with HF.^51,52,53,54^ Perhaps more important than cardiology involvement are care processes such as optimizing guideline-directed medical therapy, providing counseling on self-care, and ensuring appropriate postdischarge follow-up. Rather than efforts to increase cardiology involvement (especially given shortages in select areas), it may be more fruitful to develop strategies that can facilitate these key care processes to ensure the highest quality of care to all patients regardless of SDOH, such as low income.

Our findings raise some questions related to a recent call for elicitation and documentation of SDOH experienced by adults with chronic diseases, including HF.^47^ Given the ubiquitous role of the electronic medical record in patient care, some have advocated for incorporating SDOH fields into the electronic medical record as a strategy to improve the care of vulnerable populations.^47,55^ On the one hand, identifying adults with SDOH, such as low income, is important to identify a subpopulation at particularly high risk for poor outcomes. On the other hand, identification of SDOH could lead to implicit bias that negatively affects care provision. Consequently, it is critical that efforts intended to increase elicitation and documentation of SDOH be paired with strategies to address implicit bias from the health care system. Some previously described strategies to address implicit bias among clinicians in the health care system include implicit bias training, increasing diversity among health care workers, and partnering with community advocates.^46,47,48,56,57,58,59^ Clearly, effort is needed to routinely incorporate these strategies as a means to address the multitiered effects of SDOH.

### Strengths and Limitations

Our study has several strengths. First, we examined a geographically diverse cohort that included data on 9 SDOH that map onto an existing Healthy People 2030 framework.^37^ In addition, we had medical record–level data at the time of the HF hospitalization, which included multiple factors that could have impacted cardiologist involvement; this allowed us to adjust for several important confounders including HF severity. There are also some important limitations to note. The study sample included White and Black participants, but did not include other minoritized populations. Future studies are needed to understand the impact of SDOH on these groups. Although we examined 9 different SDOH, we did not conduct a detailed examination of other relevant factors, such as health literacy, medication adherence, health insurance, or frailty. We also did not have data to evaluate whether nonprofit or for-profit hospital status was associated with the findings. Although we had medical record–level data, involvement of a cardiologist may not have been clear in all scenarios, which could have led to misclassification bias. We also did not have hospital-level data on policies and practices for assigning patients to cardiology care, which could either reduce disparities by removing implicit bias in individual decision making or inadvertently increase disparities (eg, if people with comorbid diabetes and chronic kidney disease were directed to care by noncardiologists, there may be less cardiology involvement for Black individuals with HF). We also did not know about the patient’s care goals during the hospitalization; for example, we did not know which patients opted for comfort measures. Before deciding on pursuing comfort measures, patients and their families often want to be sure that all reasonable options have been exhausted, which may best be conveyed by a cardiologist who is trained in knowing the details of therapeutic options (pharmacologic and device-based) for advanced cardiovascular disease. Thus, the observations here likely apply to patients hospitalized for HF with a wide range of care goals.

## Conclusions

We found that low income was inversely associated with cardiologist involvement during hospitalization after adjusting for potential confounders. This suggests that certain social factors may bias the care provided to patients hospitalized for HF, calling attention to a potentially important source of disparity in care.

## Notes

Supplement 1. **eTable 1.** Collinearity for Candidate Social Determinants of Health **eTable 2. **Participant Characteristics Stratified by Cardiology Involvement **eFigure 1.** Healthy People 2030 Approach to Social Determinants of Health **eFigure 2.** Inclusion/Exclusion Cascade

Supplement 2.
Data Sharing Statement
