# Supplementary material for: Social Determinants of Health and Cardiologist Involvement in the Care of Adults Hospitalized for Heart Failure
Source: JAMA Netw Open. 2023 Nov 20;6(11):e2344070. doi: 10.1001/jamanetworkopen.2023.44070 (PMC10660170; doi:10.1001/jamanetworkopen.2023.44070)
Supplement: Supplement 1. — eTable 1. Collinearity for Candidate Social Determinants of Health eTable 2. Participant Characteristics Stratified by Cardiology Involvement eFigure 1. Healthy People 2030 Approach to Social Determinants of Health eFigure 2. Inclusion/Exclusion Cascade [file jamanetwopen-e2344070-s001.pdf]

Supplemental Online Content

Zhang DT, Onyebeke C, Nahid M, et al. Social determinants of health and cardiologist involvement in the care of adults hospitalized for heart failure. *JAMA Netw Open*. 2023;6(11):e2344070. doi:10.1001/jamanetworkopen.2023.44070

eTable 1. Collinearity for Candidate Social Determinants of Health
eTable 2. Participant Characteristics Stratified by Cardiology Involvement
eFigure 1. Healthy People 2030 Approach to Social Determinants of Health
eFigure 2. Inclusion/Exclusion Cascade

This supplemental material has been provided by the authors to give readers additional information about their work.

© 2023 Zhang DT et al. *JAMA Network Open*.

eTable 1: Collinearity for Candidate Social Determinants of Health

Phi correlation coefficients	Black race	Social isolation	No social network	Education < high school	Income < $35K	Rural residence	Zip code poverty > 25%	HPSA	State with poor healthcare infrastructure
Black race	---	0.08	0.04	0.21	0.24	0.06	0.31	0.11	0.06
Social isolation		---	0.04	0.04	0.08	0.03	0.03	0.02	0.03
No social network			---	0.03	0.03	0.01	0.02	0.03	0.04
Education < high school				---	0.32	0.02	0.13	0.04	0.08
Income < $35K					---	0.03	0.19	0.02	0.06
Rural residence						---	0.02	0.08	0.05
Zip code poverty > 25%							---	0.14	0.17
HPSA								---	0.10

© 2023 Zhang DT et al. *JAMA Network Open*.

State with poor healthcare infrastructure									---

HPSA = Health Professional Shortage Areas

© 2023 Zhang DT et al. *JAMA Network Open*.

eTable 2: Participant Characteristics Stratified by Cardiology Involvement

Characteristic, n (%)	n	Cardiology Involvement (n=751)	No Cardiology Involvement (n=249)	p-value
Age in years, median (IQR)	1000	77.26 (70.94, 83.58)	78.83 (72.62, 85.79)	0.028
Black race	1000	305 (40.6%)	109 (43.8%)	0.38
White race	1000	446 (59.4%)	140 (56.2%)	
Female	1000	345 (45.9%)	134 (53.8%)	0.031
Male	1000	406 (54.1%)	115 (46.2%)	
Region	1000			0.85
Stroke Buckle (153-county region in Georgia, North Carolina, and South Carolina)	1000	164 (21.8%)	54 (21.7%)	
Stroke Belt (Alabama, Arkansas, Georgia, Louisiana, Mississippi,		269 (35.8%)	94 (37.8%)	

© 2023 Zhang DT et al. *JAMA Network Open*.

North Carolina, South Carolina, and Tennessee)				
Rest of the contiguous United States		318 (42.3%)	101 (40.6%)	
SDOH				
Low annual household income	876	350 (53.0%)	142 (65.7%)	0.001
Living in rural areas	900	18 (2.7%)	7 (3.1%)	0.74
Living in a zip code with high poverty	991	159 (21.4%)	59 (23.4%)	0.41
Living in a Health Professional Shortage Area	1000	329 (43.8%)	102 (41.0%)	0.43
Poor public health infrastructure	1000	294 (39.1%)	90 (36.1%)	0.4
Social isolation	934	88 (12.6%)	40 (17.2%)	0.076
Social network	979	100 (13.6%)	38 (15.4%)	0.48
Low educational attainment	1000	126 (16.8%)	49 (19.7%)	0.30
HF characteristics				

© 2023 Zhang DT et al. *JAMA Network Open*.

New York Heart Association Class	763			0.67
I		37 (6.4%)	17 (9.0%)	
II		180 (31.3%)	57 (30.3%)	
III		231 (40.2%)	72 (38.3%)	
IV		127 (22.1%)	42 (22.3%)	
HFpEF	758	249 (41.1%)	87 (55.4%)	0.002
GWTG-HF Risk Score, median (IQR)	981	2.0 (2.0, 2.0)	2.0 (1.0, 2.0)	0.60
Length of stay in days, median (IQR)	996	5.0 (3.0, 8.0)	3.0 (2.0, 6.0)	<0.001
ICU stay	995	180 (24.1%)	37 (14.9%)	0.002
In-hospital cardiac arrest	996	30 (4.0%)	10 (4.0%)	1.00
Comorbid conditions				
Coronary artery disease	1000	601 (80.0%)	154 (61.8%)	<0.001
Arrhythmia	1000	340 (45.3%)	103 (41.4%)	0.28
Number of non-cardiac comorbidities, median (IQR)	1000	5.0 (4.0, 7.0)	5.0 (3.0, 7.0)	0.48

© 2023 Zhang DT et al. *JAMA Network Open*.

Diabetes	1000	386 (51.4%)	117 (47%)	0.23
Stroke	1000	192 (25.6%)	97 (26.9%)	0.85
Hospital characteristics				
Total number of hospital beds, median (IQR)	976	383 (230, 592)	335 (235, 592)	0.44
Presence of cardiac ICU	933	479 (68.2%)	174 (63.6%)	0.20
Academic hospital	945	439 (61.9%)	132 (55.9%)	0.10

IQR = interquartile range

HF = heart failure

HFpEF = heart failure with preserved ejection fraction

GWTG-HF = Get With The Guidelines®-Heart Failure

ICU = intensive care unit

© 2023 Zhang DT et al. *JAMA Network Open*.

eFigure 1: Healthy People 2030 Approach to Social Determinants of Health[37] (re-published from Sterling et al.,[25] with permission).

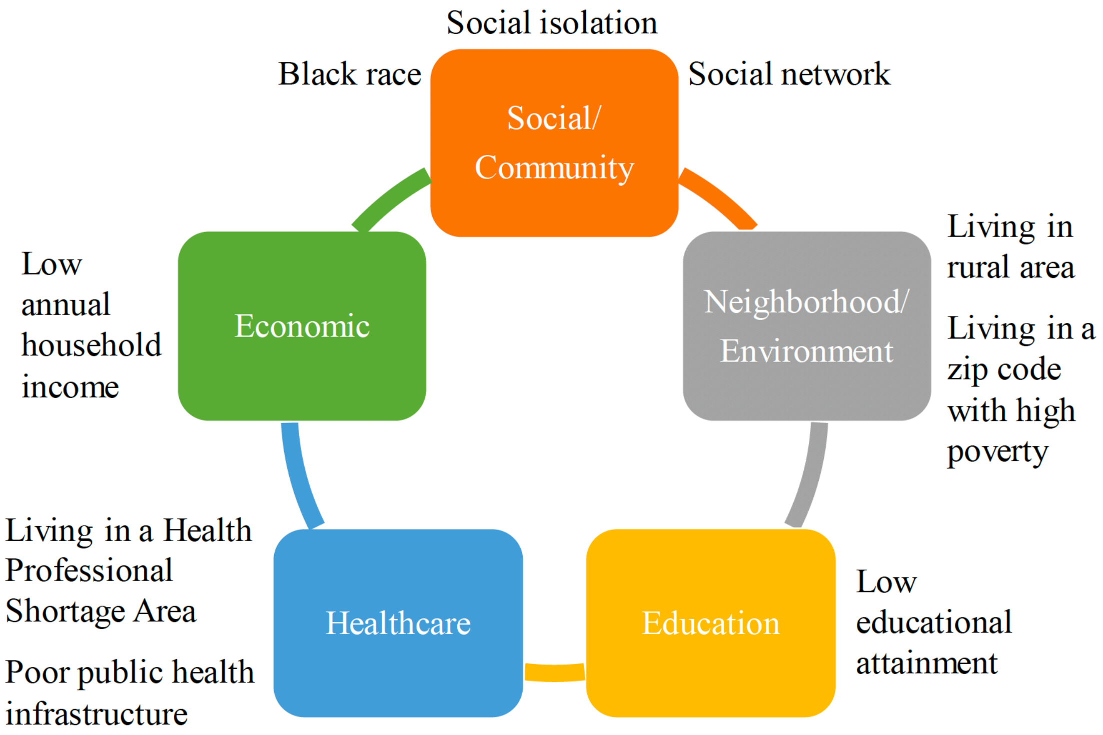

© 2023 Zhang DT et al. *JAMA Network Open*.

eFigure 2: Inclusion/Exclusion Cascade

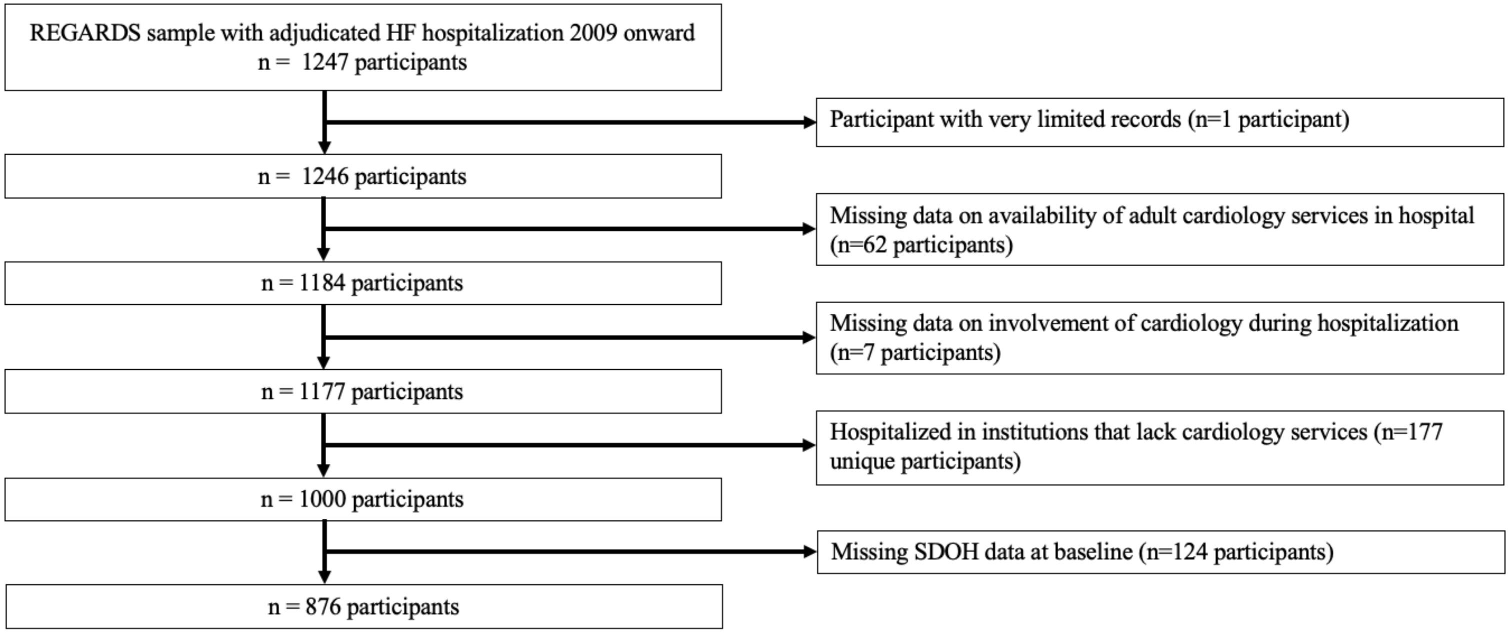

© 2023 Zhang DT et al. *JAMA Network Open*.
