# Supplementary material for: Social Determinants of Health and Cardiologist Involvement in the Care of Adults Hospitalized for Heart Failure
Source: JAMA Netw Open. 2023 Nov 20;6(11):e2344070. doi: 10.1001/jamanetworkopen.2023.44070 (PMC10660170; doi:10.1001/jamanetworkopen.2023.44070)
Supplement: Supplement 2. — Data Sharing Statement [file jamanetwopen-e2344070-s002.pdf]

Data Sharing Statement

Zhang. Social Determinants of Health and Cardiologist Involvement in the Care of Adults Hospitalized for Heart Failure. *JAMA Netw Open*. Published November 20, 2023. doi:10.1001/jamanetworkopen.2023.44070

Data
Data available: No
